# Human Adenovirus B55 Infection in Patient without Recent Travel History, France

**DOI:** 10.3201/eid3104.241936

**Published:** 2025-04

**Authors:** Menel Mohamedi, Marion Dutkiewicz, Clara Richard de Vesvrotte, Linda Feghoul, Abdeljalil Senhaji Rachik, Séverine Mercier-Delarue, Baptiste Hervier, Jérôme LeGoff, Maud Salmona

**Affiliations:** Virology Department, AP-HP, Hôpital Saint-Louis, Paris, France (M. Mohamedi, M. Dutkiewicz, L. Feghoul, A.S. Rachik, S. Mercier-Delarue, J. LeGoff, M. Salmona); Department of Internal Medicine, AP-HP, Hôpital Saint-Louis, Paris (C.R. de Vesvrotte, B. Hervier); Inserm UMR-S 976, INSIGHT Team, University Paris Cité, Paris (L. Feghoul, J. LeGoff, M. Salmona); Inserm UMR-S 976, HIPI, Université Paris Cité, Paris (B. Hervier)

**Keywords:** human mastadenovirus, B55, respiratory infections, bacteria, whole genome sequencing, phylogenetic analysis, France

## Abstract

We report a rare case of pneumonia caused by human mastadenovirus (HAdV) B55 in France in a patient without recent travel history. HAdV-B55 infection was identified retrospectively after being detected in feces during an investigation for concomitant diarrhea. This case suggests possible silent endemic circulation of HAdV-B55 in France.

Human adenovirus (HAdV) is a nonenveloped DNA mastadenovirus classified into 7 species (HAdV-A–G) with >100 identified genotypes (HAdV Working Group, http://hadvwg.gmu.edu). HAdV diversity arises from mutations or recombinations of different viral strains (*1*).

HAdV species B genotype 55 (HAdV-B55) is a recombinant virus derived from HAdV-B11 and HAdV-B14. HAdV-B55 has emerged as a major acute respiratory disease pathogen, and endemic circulation has been reported in China and South Korea (*2*). We report a case of pneumonia caused by HAdV-B55 in France.

A 51-year-old woman was hospitalized in April 2024 for febrile emesis and cough. She had no peculiar medical history, was on no medications, and was an occasional smoker. At admission to the emergency department on day 1 (D1), she was febrile at 39°C and experiencing continuous emesis. Her symptoms had started 5 days before and she had experienced no improvement, preventing her from maintaining hydration or alimentation. One episode of diarrhea had occurred 3 days previously without relapse. The patient had no recent travel, did not work in the tourism sector, and resided in a nontouristic area of Paris with her son, who had experienced influenza-like symptoms and fever 1 week earlier. No other potential exposure was identified.

Biologic testing on D1 indicated an inflammatory syndrome with elevated C-reactive protein at 135 mg/L (reference <5 mg/L) and hepatic cytolysis (aspartate aminotransferase 131 U/L [reference <32 U/L] and alanine aminotransferase 124 U/L [reference <33 U/L]). Results of blood cultures and nasopharyngeal PCR tests for SARS-CoV-2, influenza A and B, and respiratory syncytial virus were negative. Pulmonary examination found crackles in the right basilar area. A thoraco-abdomino-pelvic computed tomography scan revealed a right lower lobe consolidation. A probabilistic antibiotic therapy with amoxicillin/clavulanic acid and supplemental oxygen (1 L/min) was initiated on D1 because of a peripheral capillary oxygen saturation of 94%.

On D2, results of urinary antigen tests for *Legionella pneumophila* and *Streptococcus pneumoniae* were also negative; a sputum sample did not yield any contributory results. The patient was then transferred to the internal medicine ward.

On D3, the patient developed diarrhea. Stool culture and PCR assays for norovirus and rotavirus were negative but HAdV PCR was positive at 6.31 log_10_ copies/g of feces. HAdV typing by Sanger sequencing of the hexon and fiber genes identified HAdV-B55. Because HAdV-B55 is primarily associated with respiratory infections, we tested the D1 nasopharyngeal swab using a multiplex PCR panel (BioFire Respiratory Panel 2.1plus; bioMérieux, https://www.biomerieux.com), which returned a positive HAdV result. However, the D2 sputum sample was not preserved for HAdV testing. HAdV-specific quantitative PCR confirmed HAdV with a value below the quantification threshold (<2.70 log_10_ copies/mL). Lower respiratory tract HAdV infection could not be confirmed because of the absence of tracheal aspirate or bronchoalveolar lavage. However, HAdV PCR on a D1 serum sample showed 3.96 log_10_ copies/mL, corroborating a severe infection, but low viral load and limited remaining volume prevented sequencing for genotyping. Within 3 days of admission, the patient was weaned off oxygen and experienced improvement in respiratory and digestive symptoms. Antibiotic treatment continued for 7 days, C-reactive protein decreased to 54 mg/L, and she remained apyretic. She was discharged after 4 days (Figure 1). Retrospectively, whole-genome sequencing of HAdV-B55 from the D3 stool samples using capture probes (*3*) revealed that the patient’s sequence (GenBank accession no. PQ723065) clustered with HAdV-B55 sequences from China in 2010 (GenBank accession no. JX123027) and Argentina in 2005 (GenBank accession no. JX323384) (Figure 2).

**Figure 1:**
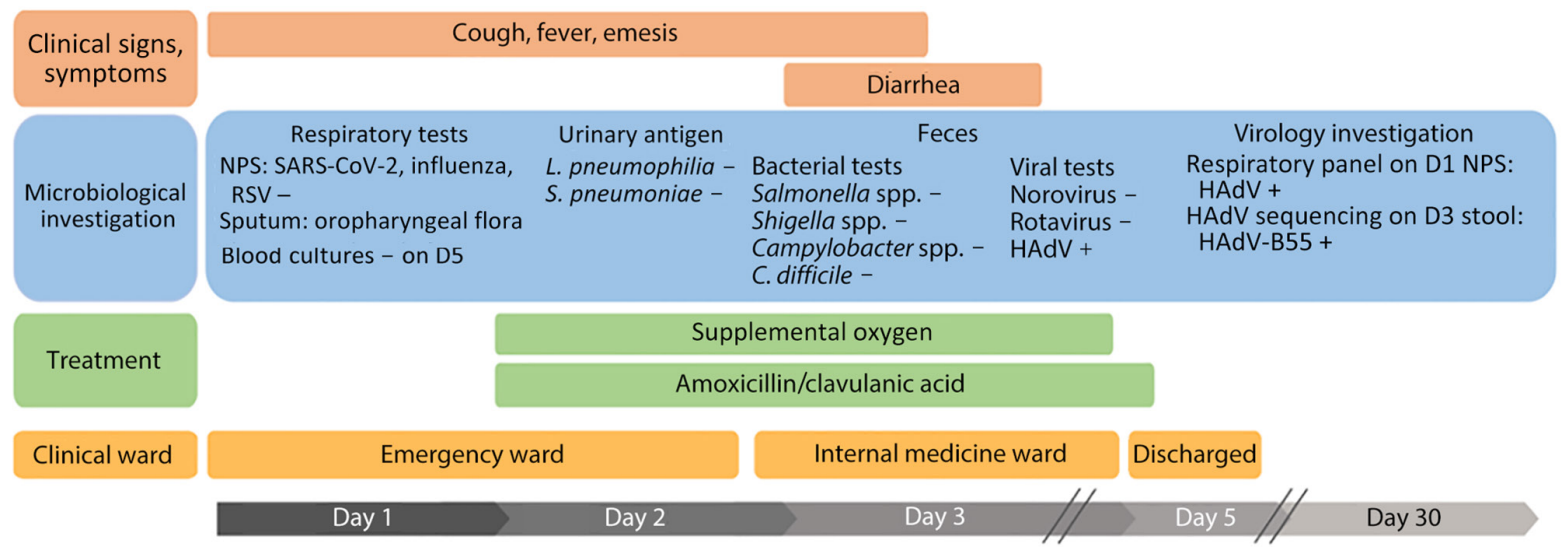
Timeline of medical care after patient’s admission to emergency department in study of HAdV-B55 infection in patient without recent travel history, France. NPS samples were negative for SARS-CoV-2, influenza, and RSV on D1, and urinary antigen tests for *Legionella pneumophila* and *Streptococcus pneumoniae* were also negative. HAdV-B55 was confirmed by whole-genome sequencing on D3 stool samples. D, day postadmission; HAdV-B55, human adenovirus B55­; NPS, nasopharyngeal swab; RSV, respiratory syncytial virus; –, negative; +, positive.

**Figure 2:**
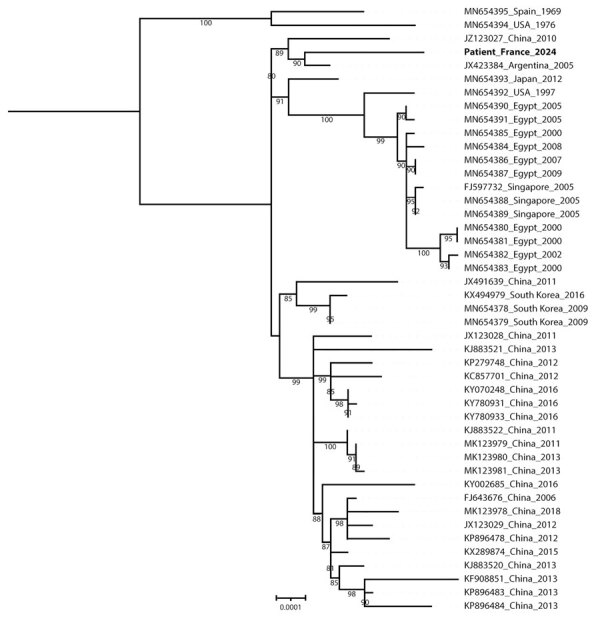
Whole-genome phylogeny of human adenovirus B55 infection in patient without recent travel history, France. The phylogenetic tree was constructed by using the maximum-likelihood method in MAFFT (https://mafft.cbrc.jp). The strains from the patient reported in this study is designated as Patient_France_2024 (GenBank accession no. PQ723065). The statistical robustness of branches was estimated by 1,000 bootstraps. Only bootstrap values >80% are indicated. GenBank accession numbers, country, and year for isolates are provided. Scale bar indicates nucleotide substitutions per site.

HAdV-B55, endemic in Asia, is poorly described in Europe (*4*). Since a 1969 outbreak in a military camp in Spain, HAdV-B55 has been reported only once in Germany (2004**)** and once in France (2014) (*5*–*7*).

In this case, the absence of recent travel suggests potential circulation of HAdV-B55 in France. Phylogenetic analysis showed that the patient’s strain is closely related to strains from China (2010) and Argentina (2005), suggesting global circulation and a broader transmission network. The China (2010) strain appears minor in Asia, and is represented by only a single sequence within this cluster.

HAdV-B55 was diagnosed atypically in this case, because HAdV was not initially tested in the respiratory sample. Instead, the virus was detected in stool after symptoms of diarrhea and vomiting. To date, only 1 study (*8*) has reported low-level HAdV-B55 (based on hexon sequencing) in stool samples from a patient in Korea with gastrointestinal and respiratory symptoms, where stool was the only specimen analyzed. Although most studies on HAdV-B55 focus on respiratory symptoms, some have reported gastrointestinal manifestations, such as vomiting and diarrhea, alongside respiratory infections (*9*,*10*). Considering a severe infection with no clear etiology, a broader viral investigation should have been conducted earlier on the D1 nasopharyngeal swab or D2 sputum. Although HAdV-B55 detection in stool suggests disease association, serum genotyping would have been more informative but was limited by low viral load and sample volume. 

In summary, this case suggests a potential underestimation of HAdV-B55 circulation in France. In addition, it underscores the need for a broader diagnostic approach to detect atypical cases and better assess the true incidence of HAdV-B55.
